# Correction to: ‘New theropod (Tetanurae: Avetheropoda) material from the ‘mid’-Cretaceous Griman Greek Formation at Lightning Ridge, New South Wales, Australia’

**DOI:** 10.1098/rsos.190230

**Published:** 2019-02-20

**Authors:** Tom Brougham, Elizabeth T. Smith, Phil R. Bell

*R. Soc. open sci.* **6**, 180826. (Published 30 January 2019). (doi:10.1098/rsos.180826)

The title of the article, ‘New theropod (Tetanurae: Avetheropoda) material from the ‘mid’-Cretaceous Griman Greek Formation at Lightning Ridge, New South Wales, Australia’, should read ‘New theropod (Tetanurae: Avetheropoda) material from the ‘mid’-Cretaceous Griman Creek Formation at Lightning Ridge, New South Wales, Australia’.

